# Tuberculosis case notifications in Malawi have strong seasonal and weather-related trends

**DOI:** 10.1038/s41598-021-84124-w

**Published:** 2021-02-25

**Authors:** Amir Kirolos, Deus Thindwa, McEwen Khundi, Rachael M. Burke, Marc Y. R. Henrion, Itaru Nakamura, Titus H. Divala, Marriott Nliwasa, Elizabeth L. Corbett, Peter MacPherson

**Affiliations:** 1 Department of Clinical Infection, Microbiology & Immunology, Institute of Infection, Veterinary & Ecological Sciences, University of Liverpool, Liverpool, UK; 2 Department of Infectious Disease Epidemiology, London School of Hygiene and Tropical Medicine, London, UK; 3 Malawi-Liverpool-Wellcome Trust Clinical Research Programme, Queen Elizabeth Central Hospital, PO30096, Blantyre, Malawi; 4 Clinical Research Department, London School of Hygiene and Tropical Medicine, London, UK; 5 Department of Clinical Sciences, Liverpool School of Tropical Medicine, Liverpool, UK; 6 Department of Infectious Diseases, Tokyo Medical University Hospital, Tokyo, Japan; 7 Helse Nord TB Initiative, College of Medicine, University of Malawi, Blantyre, Malawi

**Keywords:** Medical research, Epidemiology

## Abstract

Seasonal trends in tuberculosis (TB) notifications have been observed in several countries but are poorly understood. Explanatory factors may include weather, indoor crowding, seasonal respiratory infections and migration. Using enhanced citywide TB surveillance data collected over nine years in Blantyre, Malawi, we set out to investigate how weather and seasonality affect temporal trends in TB case notification rates (CNRs) across different demographic groups. We used data from prospective enhanced surveillance between April 2011 and December 2018, which systematically collected age, HIV status, sex and case notification dates for all registering TB cases in Blantyre. We retrieved temperature and rainfall data from the Global Surface Summary of the Day weather station database. We calculated weekly trends in TB CNRs, rainfall and temperature, and calculated 10-week moving averages. To investigate the associations between rainfall, temperature and TB CNRs, we fitted generalized linear models using a distributed lag nonlinear framework. The estimated Blantyre population increased from 1,068,151 in April 2011 to 1,264,304 in December 2018, with 15,908 TB cases recorded. Overall annual TB CNRs declined from 222 to 145 per 100,000 between 2012 and 2018, with the largest declines seen in HIV-positive people and adults aged over 20 years old. TB CNRs peaks occurred with increasing temperature in September and October before the onset of increased rainfall, and later in the rainy season during January-March, after sustained rainfall. When lag between a change in weather and TB case notifications was accounted for, higher average rainfall was associated with an equivalent six weeks of relatively lower TB notification rates, whereas there were no changes in TB CNR associated with change in average temperatures. TB CNRs in Blantyre have a seasonal pattern of two cyclical peaks per year, coinciding with the start and end of the rainy season. These trends may be explained by increased transmission at certain times of the year, by limited healthcare access, by patterns of seasonal respiratory infections precipitating cough and care-seeking, or by migratory patterns related to planting and harvesting during the rainy season.

## Introduction

Tuberculosis (TB) epidemics in sub-Saharan Africa have been driven by generalised HIV epidemics, but expanded access to antiretroviral therapy (ART) has substantially reduced the overall burden of HIV-related TB ^1^. Like other respiratory infections, seasonal patterns of TB have been observed in several countries in temperate regions ^2–4^. Nevertheless, despite the high TB disease burden, few studies have investigated seasonal trends in sub-Saharan Africa. TB diagnoses in two South African studies were at their zenith between September and November ^5,6^. In a study from the Western Cape, South Africa that analysed over 100-years of TB case notification data, seasonal peaks were also found between September and November ^7^. Similarly, in Zimbabwe, laboratory-confirmed TB case notifications markedly increased between September and October ^8^. Pulmonary TB notifications among HIV-positive people in South Africa and Zimbabwe showed seasonal variations which mirrored fluctuations in clinical activity, with lower notification rates in December and rebounds in January–February ^9^.

Seasonality in TB diagnosis is poorly understood. Seasonal changes in temperature and particulate matter exposure may lead to worsening of respiratory symptoms, prompting care seeking ^10,11^. Lower temperatures and higher rainfall may increase indoor crowding and promote indoor droplet transmission ^12–14^. Outbreaks of seasonal respiratory infections such as seasonal coronavirus infections and influenza may stimulate cough or other respiratory symptoms, “unmasking” TB disease ^15^. During seasonal planting and harvesting periods, migration from urban to rural areas may inhibit access to TB diagnosis and treatment services. Because of these potentially interrelated factors, the effects of season on TB case notifications may vary across age groups, by sex, and by HIV status. As active TB often has a variable and long incubation period and prolonged periods between symptom onset and diagnosis, there may be lags between weather dynamics and TB case notification rates (CNRs).

Understanding how seasonality and weather affect patterns of TB CNRs could help generate hypotheses for the underlying causal pathways, predict temporal trends in healthcare utilisation, and develop strategies to improve access to TB diagnosis and care. Using high-resolution surveillance data collected over nine years in Blantyre, Malawi, we aimed to investigate the relationship between trends in TB CNRs and weather conditions by different demographic groups.

## Methods

### Study site and population

Blantyre is a major commercial centre in in the Southern region of Malawi, with an estimated adult HIV prevalence of 18% ^16^. Blantyre District is administratively divided into Blantyre City and Blantyre Rural, with a combined 2018 mid-year census population of 1,264,304 ^17^.

### Blantyre enhanced TB surveillance

Since 2011, the Malawi-Liverpool-Wellcome Trust (MLW) in partnership with the Malawi National TB Programme and the Blantyre District Health Office have been conducting enhanced TB surveillance in Blantyre which was designed to answer several operational and research questions ^18^. We used this prospectively collected data to conduct our analyses. In brief, people registering for TB treatment at all health facilities in Blantyre District had demographic and TB clinical characteristics recorded (initially on paper forms, and subsequently electronically) by TB Officers working for the Ministry of Health of Malawi. TB Officers initiated TB treatment, performed HIV testing and made ART referrals as appropriate in accordance with Ministry of Health Guidelines. At registration for TB treatment, a spot sputum was collected from all patients able to produce a sample and transported to the TB Research Laboratory at the College of Medicine, University of Malawi for smear and mycobacteria growth indicator tube (MGIT) culture. TB patients were geolocated to their district of residence (either within, or out of Blantyre District) by a satellite mapping system ^18^. Monthly data from enhanced TB surveillance records are reconciled with Ministry of Health TB Registers. In this study, we include TB case notifications between April 2011 and December 2018.

### Population denominators and estimation of TB case notification rates

We used data from the 2008 and 2018 Malawi National censuses to obtain mid-year population estimates for Blantyre District, stratified by age groups and sex. We used age- and sex- specific data from an HIV prevalence survey conducted in Blantyre (available at: https://github.com/petermacp/mlwdata). We applied these age- and sex- specific prevalence estimates to national census population data for those over the age of 16 to produce HIV-specific population denominators. Linear interpolation and extrapolation were used to calculate quarterly sex- and age group-specific population denominators. Time trends in TB case notification rates per 100,000 people were calculated by dividing the number of TB cases registered in each quarter-sex-age group stratum by the stratum-specific population denominator.

### Seasons and weather

Malawi has three distinct seasons: a cold-dry season with low relative humidity (approximately May–August), a hot season with low relative humidity (approximately September–November), and a rainy season with high relative humidity (approximately December–April). Daily weather data (mean temperature [°C] and total rainfall [mm]) were obtained for Chileka Weather Station in Blantyre from the Global Surface Summary of the Day database ^19^.

### Statistical analysis

We summarised the characteristics of patients initiating TB treatment in Blantyre between 2011 and 2018 using percentages, means (with standard deviations), and medians (with interquartile ranges). We plotted quarterly trends in TB case notification rates and 10-week moving averages with 95% binomial exact confidence intervals for sex-age group strata. We additionally plotted trends in 10-week moving temperature average and daily rainfall.

To investigate the associations between weather conditions and TB case notification rates, we fitted two separate generalized linear models in the distributed lag nonlinear modelling framework using the ‘dlnm’ R package ^20^. The seasonal-unadjusted reported TB notifications on week number $t$, ($X_{t}$), was assumed to follow an overdispersed Poisson distribution with mean ($\lambda_{t}$) and variance ($\phi \lambda_{t}$), where $\phi$ is an estimated overdispersion parameter (Eqs. 1 and 2).

1 $$X_{t} \sim Poisson\left( {\lambda_{t} ,\phi } \right)$$

2 $$Log\left( {\lambda_{t} } \right) = \alpha + \beta_{w} + \delta_{T} + \mathop \sum \limits_{l = 0}^{20} f.g\left( {x_{t - l} ,l} \right) + \varepsilon_{t}$$

where $\lambda_{t}$ is the mean number of reported TB notifications for week $t$ where $t = 1, \ldots , 455$ (~ 9 years of TB notifications) indexes week number, *α* is the model intercept, $\beta_{w}$ are weeks random effects to account for seasonality where, $w \approx 1, \ldots ,52$ indexes the epidemiological week, $\delta_{T}$ are years random effects to account for the long-term trend where $T \approx 1, \ldots ,8$ indexes the year number, the cross-basis function $\mathop \sum \nolimits_{l = 0}^{20} f.g\left( {x_{t - l} ,l} \right)$ is the nonlinear weather variable $f\left( x \right)$ and lag $g\left( l \right)$ natural cubic spline functions combination, with lags $l$ from 0 to 20 weeks where $x$ is the either rainfall or temperature ^21^, $\varepsilon_{t}$ are the residuals added at specific lags to correct for partial autocorrelation when substantially high.

The models were fitted to the weekly TB notifications data. Concurrently, we estimated the potential nonlinear and delayed effects of rainfall and temperature on weekly TB notifications. Rainfall, temperature and lags were captured using natural cubic spline functions to flexibly model nonlinear relationship between weather-lag and TB notifications.

The combined spline functions produced separate cross-basis matrices of rainfall-lag and temperature-lag. The first model included rainfall-lag, seasonality, long-term trend and residuals, and the second model included temperature-lag, seasonality, long-term trend, and residuals. Additionally, each model was conditioned on sex, age group, HIV status and diagnosis type specific datasets to obtain stratified estimations on CNRs of the delayed impact of rainfall and temperature.

A total of 81 potential models were generated by varying the degrees of freedom (*df*) representing each weather-lag spline function. A similar application has been described elsewhere ^22,23^. In brief, we used an optimal 3 *df* per year without an intercept each for the rainfall, temperature and lag spaces. Of the 81 potential models generated, two models with minimum Quasi-Akaike Information Criterion (QAIC) scores corresponding to *df* per year for rainfall-lag or temperature-lag were selected (Eq. 3, Supplementary Table 1) ^24^.

3 $$QAIC = - 2L\left( \theta \right) + 2\phi k$$

where $L$ is the log-likelihood of the Poisson distribution fitted model with a set of parameters ${\uptheta }$, $\phi$ is the estimated overdispersion parameter and $k$ is the number of model parameters.

For each selected model of rainfall lag and temperature lag, the temporal residual deviances, autocorrelation and partial autocorrelation were examined and adjusted to reduce partial autocorrelation at specific lags to below the pre-specified thresholds. The accuracies of model predictions for rainfall and temperature relative to the observed data were computed using the mean absolute percentage error metric (Supplementary Fig. 1) ^25,26^.

Model sensitivity analysis was conducted by examining the impact of alternative models after varying the *df* in the cross-basis functions of rainfall-lag and temperature-lag on the shapes of the weather-TB notification relationship (Supplementary Fig. 2). All analyses were conducted in R v3.2.4 ^27^, and statistical significance was set at *p* < 0.05. Data and code are available online at: https://github.com/petermacp/seasontb.

### Ethical considerations

Ethical approval was granted by the London School of Hygiene and Tropical Medicine and the College of Medicine, University of Malawi Research Ethics Committee. Participants gave oral consent to participate in TB surveillance with a waiver for written consent granted by both research ethics committees. All methods were carried out in accordance with relevant national guidelines and regulations.

## Results

### Baseline characteristics

Between 1^st^ April 2011 to 31^st^ December 2018, we recorded 15,908 TB treatment registrations in Blantyre (Table 1). Of these, 13,924 (87.6%) were new cases, 1371 (8.6%) had relapsed TB and 90 (0.5%) were retreatment cases (519 had other TB classifications; 4 missing). Ages ranged from under one to 94 years old, with a mean age of 34.1 (SD 14.4). There were 6,181 (38.9%) female and 9,727 (61.1%) male presentations, and 10,025 (63%) pulmonary and 5,880 (37%) extra-pulmonary presentations (data missing for 3 cases). 10,421 (70.3%) were HIV-positive and 4,407 (29.7%) were HIV-negative (1080 missing). Of 12,841 with results, 7,052 (54.9%) had smear/Xpert-positive TB and 5,789 (45.1%) were smear/Xpert-negative.

**Table 1:** Characteristics of Blantyre TB patients registered for treatment, April 2011 to December 2018.

	2011 (N = 1309)	2012 (N = 2464)	2013 (N = 2347)	2014 (N = 2110)	2015 (N = 1962)	2016 (N = 1957)	2017 (N = 1940)	2018 (N = 1819)	Total (N = 15,908)	*P*-value
**Season**										< 0.001
Cold, dry (May-Aug)	396 (30.3%)	864 (35.1%)	776 (33.1%)	666 (31.6%)	645 (32.9%)	616 (31.5%)	650 (33.5%)	677 (37.2%)	5290 (33.3%)	
Hot, dry (Sept-Nov)	671 (51.3%)	693 (28.1%)	564 (24.0%)	543 (25.7%)	477 (24.3%)	524 (26.8%)	493 (25.4%)	439 (24.1%)	4404 (27.7%)	
Hot, wet (Dec-Apr)	242 (18.5%)	907 (36.8%)	1007 (42.9%)	901 (42.7%)	840 (42.8%)	817 (41.7%)	797 (41.1%)	703 (38.6%)	6214 (39.1%)	
**Sex**										0.025
Female	504 (38.5%)	1008 (40.9%)	953 (40.6%)	844 (40.0%)	732 (37.3%)	721 (36.8%)	728 (37.5%)	691 (38.0%)	6181 (38.9%)	
Male	805 (61.5%)	1456 (59.1%)	1394 (59.4%)	1266 (60.0%)	1230 (62.7%)	1236 (63.2%)	1212 (62.5%)	1128 (62.0%)	9727 (61.1%)	
**Age**										< 0.001
Mean (SD)	31.8 (14.3)	33.1 (14.9)	34.1 (14.5)	34.6 (13.8)	34.6 (13.4)	34.5 (13.7)	34.4 (14.6)	35.7 (15.2)	34.1 (14.4)	
**HIV status**										< 0.001
Missing	193	234	291	159	115	50	31	7	1080	
HIV-negative	300 (26.9%)	599 (26.9%)	573 (27.9%)	599 (30.7%)	532 (28.8%)	604 (31.7%)	612 (32.1%)	588 (32.5%)	4407 (29.7%)	
HIV-positive	816 (73.1%)	1631 (73.1%)	1483 (72.1%)	1352 (69.3%)	1315 (71.2%)	1303 (68.3%)	1297 (67.9%)	1224 (67.5%)	10,421 (70.3%)	
**ART**										< 0.001
Not taking ART	894 (68.3%)	1445 (58.6%)	1200 (51.1%)	1020 (48.3%)	862 (43.9%)	792 (40.5%)	735 (37.9%)	635 (34.9%)	7583 (47.7%)	
Taking ART	415 (31.7%)	1019 (41.4%)	1147 (48.9%)	1090 (51.7%)	1100 (56.1%)	1165 (59.5%)	1205 (62.1%)	1184 (65.1%)	8325 (52.3%)	
**TB classification**										< 0.001
Missing	0	0	0	0	0	0	0	3	3	
Pulmonary TB	921 (70.4%)	1721 (69.8%)	1602 (68.3%)	1223 (58.0%)	1244 (63.4%)	1200 (61.3%)	1201 (61.9%)	913 (50.3%)	10,025 (63.0%)	
Extrapulmonary TB	388 (29.6%)	743 (30.2%)	745 (31.7%)	887 (42.0%)	718 (36.6%)	757 (38.7%)	739 (38.1%)	903 (49.7%)	5880 (37.0%)	
**TB category**										< 0.001
Missing	0	0	0	0	0	0	0	4	4	
New TB case	1190 (90.9%)	2214 (89.9%)	2049 (87.3%)	1832 (86.8%)	1701 (86.7%)	1665 (85.1%)	1675 (86.3%)	1598 (88.0%)	13,924 (87.6%)	
Relapse TB case	87 (6.6%)	180 (7.3%)	165 (7.0%)	161 (7.6%)	144 (7.3%)	189 (9.7%)	245 (12.6%)	200 (11.0%)	1371 (8.6%)	
Retreatment after default	1 (0.1%)	7 (0.3%)	7 (0.3%)	10 (0.5%)	8 (0.4%)	6 (0.3%)	6 (0.3%)	9 (0.5%)	54 (0.3%)	
Retreatment after failure	2 (0.2%)	1 (0.0%)	6 (0.3%)	6 (0.3%)	4 (0.2%)	7 (0.4%)	5 (0.3%)	5 (0.3%)	36 (0.2%)	
Other	29 (2.2%)	62 (2.5%)	120 (5.1%)	101 (4.8%)	105 (5.4%)	90 (4.6%)	9 (0.5%)	3 (0.2%)	519 (3.3%)	
**Smear or Xpert positive TB**										< 0.001
Missing	305	429	464	228	282	412	418	529	3067	
Smear/Xpert-negative	449 (44.7%)	938 (46.1%)	920 (48.9%)	941 (50.0%)	759 (45.2%)	558 (36.1%)	621 (40.8%)	603 (46.7%)	5789 (45.1%)	
Smear/Xpert-positive	555 (55.3%)	1097 (53.9%)	963 (51.1%)	941 (50.0%)	921 (54.8%)	987 (63.9%)	901 (59.2%)	687 (53.3%)	7052 (54.9%)	

### Population and TB case notification rate

**Figure 1:**
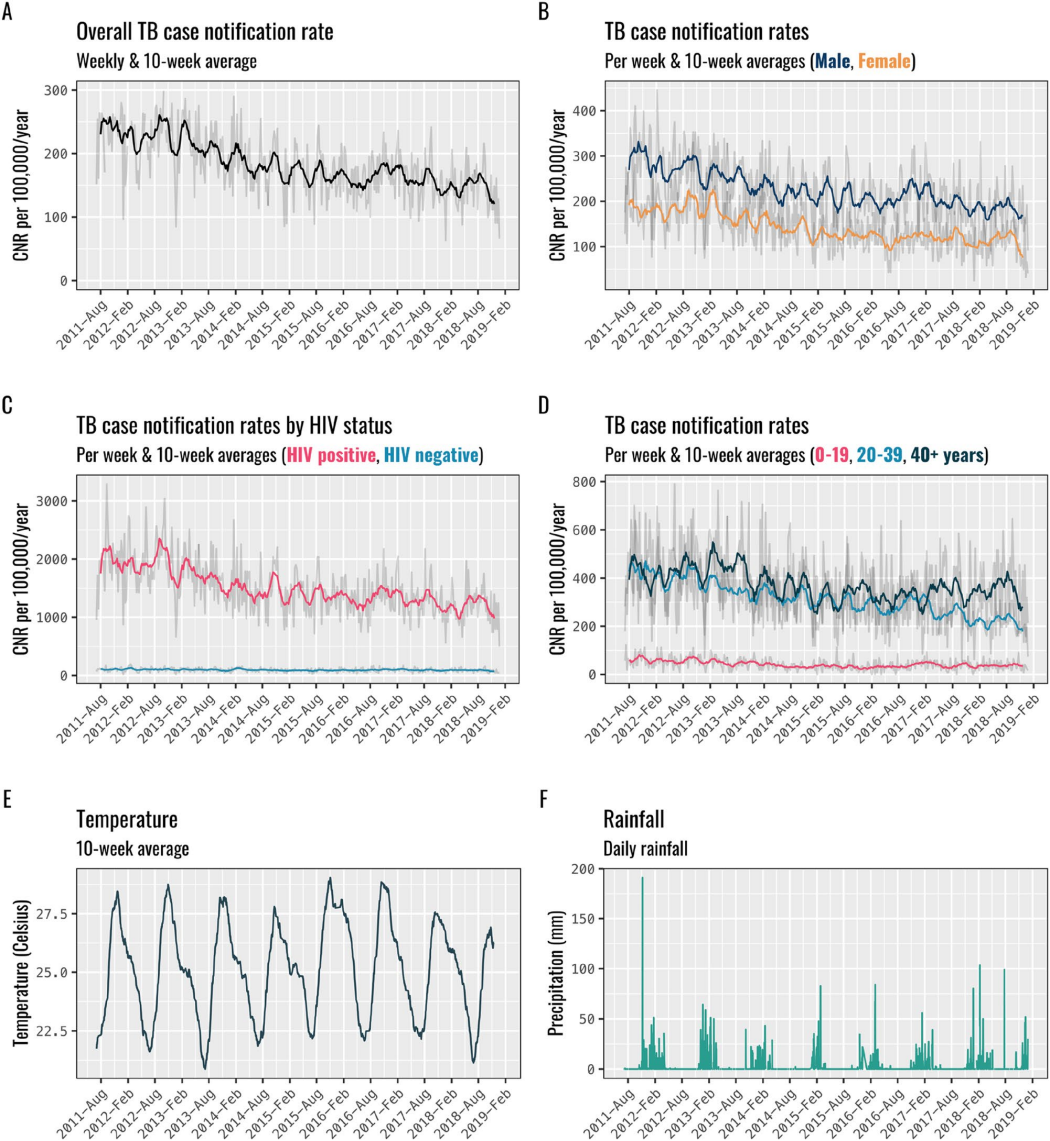
Blantyre tuberculosis case notification rates, average weekly rainfall and temperature (April 2011–December 2018).

The population of Blantyre increased from 1,068,151 to 1,264,304 between April 2011 and December 2018. There was a declining trend in TB CNRs over the study period, falling year-on-year from 222 (95% CI: 150–329) in 2012 to 145 (95% CI: 92–230) per 100,000 in 2018. TB CNRs declined among people living with HIV (PLHIV) over this time (1936 per 100,000 in 2012 to 1189 in 2018) with little change in CNRs for those who were HIV-negative. TB CNRs also declined amongst adults aged over 20 with little change in CNRs for those under 20 years (Fig. 1, Panel D).

A 10-week moving average of the TB CNRs over this period showed a pattern of peaks and troughs indicating seasonal variation in the TB CNRs (Fig. 1), with most years having two peaks. Peaks tended to coincide with increasing temperature in September and October before the onset of rains, and later in the rainy season during January and March.

Similar seasonal variation in TB CNRs was seen throughout stratified groups and did not vary based on age, sex or HIV status.

### Associations between weather and TB notifications

**Figure 2:**
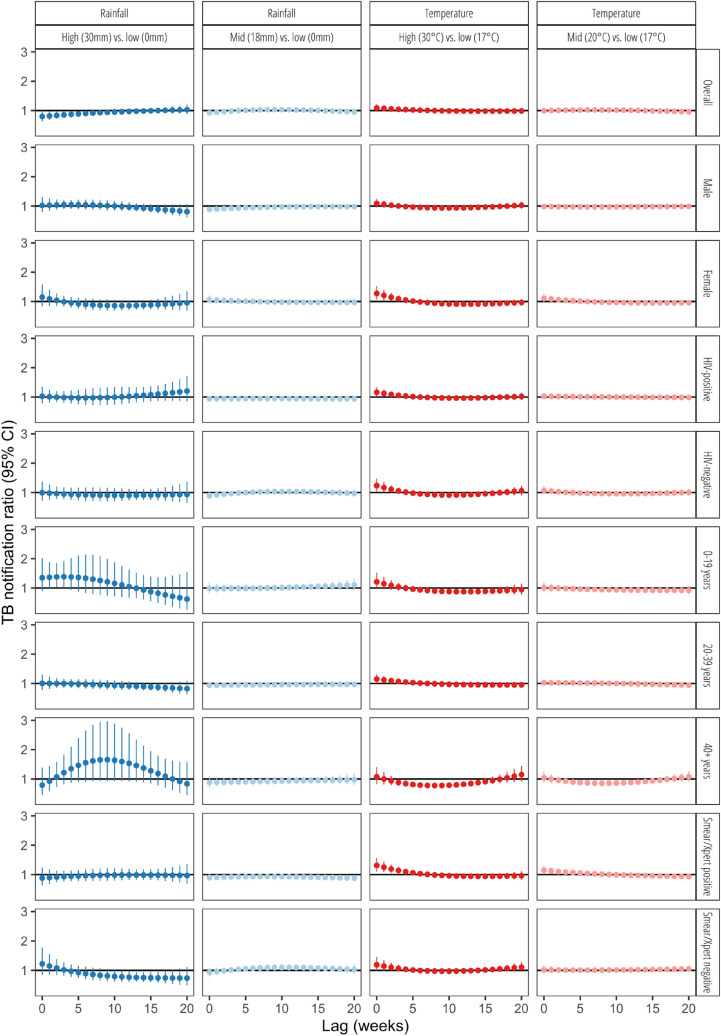
Associations between delayed TB case notifications and weekly lag in rainfall and temperature.

Figure 2 shows associations between the distributed week-lag rainfall and temperature and TB notifications, comparing conditions with no rainfall (0 mm) and low weekly average temperature (17 °C) to conditions with weekly average mid (18 mm) and high rainfall (30 mm), mid (20 °C) and high temperature (30 °C). Overall, high rainfall was significantly associated with lower TB notifications; this association was greatest in the first 6 weeks from onset of heavy rains, with an immediate maximum relative reduction of − 10% (notification relative ratio [RR] 0.90, 95%CI [0.81–0.99]). Mid or high temperatures were not associated with significant changes in relative notifications compared to low temperature.

### Rainfall and TB notifications by population subgroups

In the stratified analyses, associations between rainfall and TB notifications varied by sex, smear/Xpert status and age group (Fig. 2, Supplementary Fig. 3). Comparing weeks with mid rainfall (18 mm per day) to no rainfall, notifications among men were significantly relatively lower during weeks 0–4, and were at their lowest immediately (week 0, − 11%, RR: 0.89, 95%CI: 0.79–0.99]). Individuals with smear/Xpert positive results had reduced TB notifications between weeks 12 and 20 after the onset of mid rainfall, and lowest at  − 12% in week 10 (RR: 0.88, 95%CI [0.78–0.99]). However, adults over 40 years old had increased TB notifications between 2 and 17 weeks after the onset of heavy rainfall, peaking at + 66% in week 9, although this was not statistically significant (RR: 1.66, 95%CI [0.93–2.97]).

### Temperature and TB notifications by population subgroups

Associations between temperature and TB notifications varied significantly by age group, sex, HIV status and smear/Xpert status (Fig. 2, Supplementary Fig. 3). From 4 to 14 weeks after onset of high and mid weekly temperatures (and consistent with the relative rise in notifications seen during the same lag period after the onset of heavy rains), adults over 40 years old had significantly lower notifications, greatest at lag week 8 (high vs. low temperature: − 22%, RR 0.78, 95%CI [0.68–0.88]; mid vs. low temperature: − 15% (RR 0.85, 95%CI [0.79–0.94]). Younger people (aged 0–19 years) had significant lower notifications during high temperature weeks, commencing after 10–15 weeks lags, with the greatest reduction of − 12% at week 12 (RR 0.88, 95%CI [0.79–0.97]).

Women (+ 28%, RR 1.28, 95%CI [1.07–1.52], HIV-negative (+ 24%, RR 1.24, 95%CI [1.03–1.48]), PLHIV (+ 16%, RR 1.16, 95%CI [1.01–1.33]), and adults aged 20–39 years old (+ 15%, RR 1.15, 95%CI [1.01–1.31]) had significant and immediate increases in TB notifications with the onset of high temperature weeks. Notifications among individuals with smear/Xpert negative (+ 24%, RR: 1.24, 95%CI [1.03–1.49], high vs. low temp weeks) and (+ 16%, RR: 1.16, 95%CI [1.01–1.32], mid vs. low temp weeks) were significantly increased during weeks 17–20 after onset of respective temperatures, peaking at week 20.

### Sensitivity analysis

In a sensitivity analysis, an increase in the *df* from 3 to 5 in the lag space produced less smooth curves and large confidence intervals of the delayed effects of rainfall and temperature on TB notifications, likely due to overfitting and lack of data points with increasing knots (more parameters). An increase in the *df* from 4 to 5 in the rainfall and temperature spaces produced smoother curves with evidence of better fit, which was more striking for the temperature space. Regardless of the model being sensitive to changes in the higher *df* in the lag, rainfall and temperature spaces, our overall estimations with optimal *dfs* produced smoother curves, and our final models have a low mean absolute percentage error of around 17% (> 80% accuracy) (Supplementary Fig. 2).

## Discussion

Using data from enhanced citywide prospective surveillance linked to population census denominators, we observed a consistent pattern in TB case notification rates of two cyclical peaks in approximately September to October (just before onset of heavy rains) and January to March (during the rainy season) each year. There was an encouraging trend of decreasing TB case notifications over the study period, greatest among PLHIV and likely due to improving HIV/TB care. Our season-adjusted short-term estimates showed that the onset of heavy rain had an immediate effect of reducing TB notifications overall, and among men and those with smear/Xpert positive TB. The beginning of hot weather was an important determinant of increased TB notifications among women, PLHIV, HIV-negative people, adults aged 20–39 years, and for those with smear/Xpert negative status at extended lag. In contrast, TB notifications reduced for children (0–19 years) and older adults (> 40 years) at longer week-lags during high temperature. Taken together, these findings indicate that weather conditions are associated with changes in TB case notifications and exert differential influence on particular population groups.

We postulate an interlinked set of determinants to explain weather-TB notification dynamics in Blantyre. In Malawi and other sub-Saharan countries, many people migrate from urban areas during onset of the rainy season to plant crops, with older adults more engaged in subsistence and commercial farming activities ^28^. This period of rainfall onset around November concedes with the end of hot temperatures and is consistent with our results of reduced overall TB notifications, and among males, children and older adults. Being out of the city and prioritising farming may imply that they are less likely or able to access healthcare or TB diagnostic services. Care-seeking may not happen until after the planting period is completed. With adults moving to farmlands, children are also less likely to be identified with TB-like symptoms in the absence of caregivers. It may also mean that household crowding is reduced during this period resulting in even lower transmission events of TB and related respiratory infections leading to reduced care seeking. With the usual onset of heavy rains, access to clinics (both for patients and health workers) may be challenging, with substandard roads, flooding and storms making roads treacherous and limiting transport options. Mobility data could be used to further understand seasonal migration patterns and evaluate the relative contributions of movement for seasonal planting and harvesting on case notifications. In addition to migration, detailed examination of the interactions between public holidays, weekend days and weather could further shed light on how travel patterns influence care seeking for TB, particularly during December where clinical services may be reduced. Individuals with smear/Xpert positive status had reduced notifications immediately from heavy rain onset. It is likely that a male sex could be driving this phenomenon as they had higher notifications than females. Additionally, men have substantially longer delays in initiating care-seeking for TB compared to women ^29^. However, without the means to examine interactions between sex, TB diagnosis and weather in this modelling framework, this observation remains inconclusive.

Relatively high indoor crowding during rainy season may increase TB transmission particularly in densely populated slums ^30^. Indoor crowding is probably highest during the cold-dry season, around May–July, which lies between end of the rainy season and beginning of hot season in Malawi. In Cameroon, TB cases increased during rainy seasons ^31^. Combined with high incidence of other seasonal respiratory infections, this may result in worsening symptoms potentially resulting in seasonal peak notifications during the rainy season in January to March ^32^. Esmail et al. postulated that bouts of cough due to infections other than TB (“unrelated cough”) may promote TB transmission and increase subjective awareness of previous subclinical TB symptoms, increasing the likelihood sputum positivity, and of care-seeking and TB diagnosis ^33^. Respiratory infections such as seasonal coronavirus and pneumococcal pneumonia tend to peak after October in the Southern Hemisphere and may cause or worsen a cough and prompt care seeking ^15,34^. Influenza predominantly circulates between January and April in Africa, coinciding with the peak in TB case notifications late in the rainy season ^35^. Thus, the estimated higher TB notifications in different groups at the beginning of hot season is likely driven by prior transmission of respiratory infections during cold season.

Although some of our results are similar to what others have previously reported on the influence of seasonality and weather on TB notifications, some differences and limitations exist. For instance, in Bangladesh, TB incidence was significantly associated with both lower rainfall and temperature ^36^. Given the differential effects of weather on TB CNRs by setting, future studies could collate all the estimates of the temporal effects of weather on TB CNRs globally using routine programmatic notification data to further understand relationship differences. These studies may also be able to measure the impact of climate change on seasonal TB changes by surveillance over longer periods of time.

We suggest that the results from this study can be used to plan service needs in Malawi by anticipating higher service use between November and March. Local health services can also use these data to address potential service issues which contribute to delayed diagnosis of TB, for example after heavy rainfall, or due to changes in clinical service provision at certain times of the year. Qualitative research could improve our understanding of behavioural factors which influence care seeking and seasonality and can be used to target specific population groups at certain times of the year when delays in care seeking are anticipated. Mobility studies could use cell phone data in Malawi to look at how migration affects care seeking and presentation to health services seasonally.

There were several limitations to this study. Although reconciliation of the study electronic registers and the national TB programme registers has consistently demonstrated high concordance, some TB notifications may not have been registered for various administrative reasons and changes in registration practices may have occurred over time. We obtained weather data from a single weather station whereas weather patterns do vary across Blantyre, and our analysis could have benefited from incorporation of local neighbourhood weather data. Data on other important covariates including particulate matter levels, health service utilisation data, and migration and mobility were not available. Although we carefully selected models to best predict the temporal effects of weather on TB notifications, these associations are ecological and cannot be interpreted as causal. Finally, we were not able to investigate the interactions between different covariates and lagged weather conditions due to limitations of the modelling framework software.

In conclusion, TB CNRs in Blantyre are seasonal with peaks at the start and end of the rainy season, and are significantly influenced by weather conditions, particularly heavy rainfall and extreme temperatures. Variations in TB CNR due to seasonality and weather could be mediated by increased transmission due to indoor overcrowding, limited healthcare access, patterns of seasonal respiratory infections precipitating cough and care-seeking, or migratory patterns related to planting and harvesting during rainy season.

## Supplementary Information

Supplementary Information
