# Supplementary material for: Tuberculosis case notifications in Malawi have strong seasonal and weather-related trends
Source: Sci Rep. 2021 Feb 25;11:4621. doi: 10.1038/s41598-021-84124-w (PMC7907065; doi:10.1038/s41598-021-84124-w)
Supplement: Supplementary file 1 — Supplementary Information [file 41598_2021_84124_MOESM1_ESM.docx]

**Supplementary Table S1. Natural cubic spline functions for weekly mean rainfall and temperature, and lag.**

| **Model**  **no** | **Lag *(df)*** | **Rainfall (*df)*** | **Temperature *(df)*** | **QAIC (rainfall)** | **QAIC (temperature)** | **QAIC (rainfall & temperature)** |
| --- | --- | --- | --- | --- | --- | --- |
| 1 | 3 | 3 | 3 | 1474.744* | 1496.242* | 1544.778* |
| 2 | 4 | 3 | 3 | 1493.405 | 1543.736 | 1588.154 |
| 3 | 5 | 3 | 3 | 1499.362 | 1543.200 | 1584.064 |
| 4 | 3 | 4 | 3 | 1481.011 | 1496.242 | 1553.102 |
| 5 | 4 | 4 | 3 | 1507.072 | 1543.736 | 1603.645 |
| 6 | 5 | 4 | 3 | 1512.846 | 1543.200 | 1599.677 |
| 7 | 3 | 5 | 3 | 1487.595 | 1496.242 | 1553.114 |
| 8 | 4 | 5 | 3 | 1520.859 | 1543.736 | 1607.244 |
| 9 | 5 | 5 | 3 | 1525.261 | 1543.200 | 1601.280 |
| 10 | 3 | 3 | 4 | 1474.744 | 1496.954 | 1547.544 |
| 11 | 4 | 3 | 4 | 1493.405 | 1545.320 | 1593.432 |
| 12 | 5 | 3 | 4 | 1499.362 | 1545.976 | 1591.291 |
| 13 | 3 | 4 | 4 | 1481.011 | 1496.954 | 1560.095 |
| 14 | 4 | 4 | 4 | 1507.072 | 1545.320 | 1615.268 |
| 15 | 5 | 4 | 4 | 1512.846 | 1545.976 | 1613.226 |
| 16 | 3 | 5 | 4 | 1487.595 | 1496.954 | 1561.723 |
| 17 | 4 | 5 | 4 | 1520.859 | 1545.320 | 1622.134 |
| 18 | 5 | 5 | 4 | 1525.261 | 1545.976 | 1619.052 |
| 19 | 3 | 3 | 5 | 1474.744 | 1558.252 | 1605.070 |
| 20 | 4 | 3 | 5 | 1493.405 | 1601.826 | 1647.801 |
| 21 | 5 | 3 | 5 | 1499.362 | 1598.002 | 1641.562 |
| 22 | 3 | 4 | 5 | 1481.011 | 1558.252 | 1616.131 |
| 23 | 4 | 4 | 5 | 1507.072 | 1601.826 | 1666.718 |
| 24 | 5 | 4 | 5 | 1512.846 | 1598.002 | 1660.037 |
| 25 | 3 | 5 | 5 | 1487.595 | 1558.252 | 1616.731 |
| 26 | 4 | 5 | 5 | 1520.859 | 1601.826 | 1673.319 |
| 27 | 5 | 5 | 5 | 1525.261 | 1598.002 | 1664.834 |

Degrees of freedom (df) associated with cross-basis matrices in the weather-lag spaces, and values for the Quasi-Akaike Information Criterion (QAIC) for the alternative models for the association between rainfall, temperature and TB notifications from year 2011 to 2018.

**Supplementary Figure S1. Sensitivity analysis.**

| 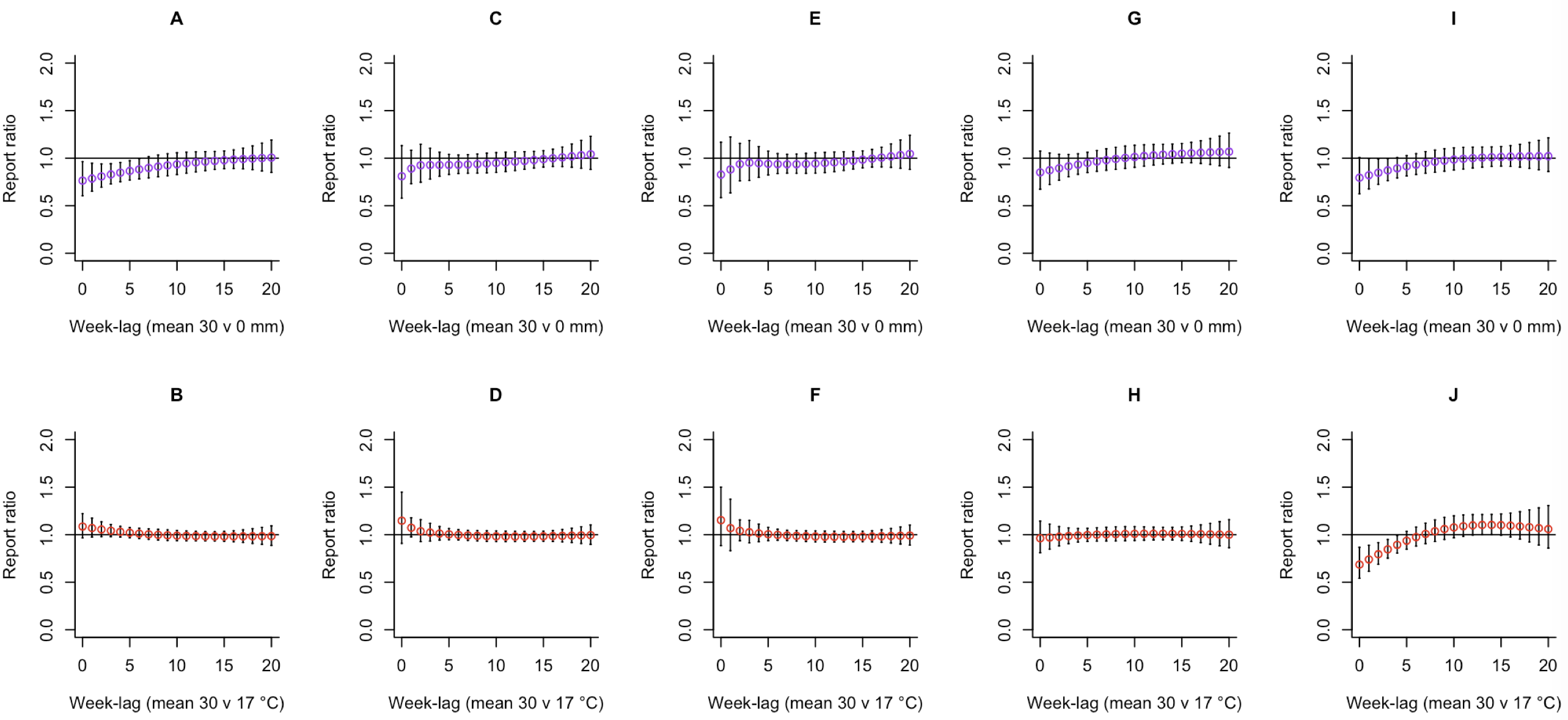 |
| --- |
| The effects on TB notifications of weekly average of 30 mm rainfall compared to 0 mm and weekly average of 30 °C temperature compared to 17 °C along the lags, modified by increasing the degrees of freedom (df) in the lag space from (df=3; model.no=1;A,B) to (df=4; model.no=2;C,D) to (df=5; model.no=3;E,F), in the rainfall space from (df=4; model.no=4;G) to (df=5; model.no=7;I), in the temperature space from (df=4; model.no=10;H) to (df=5; model.no=19;J) |

**Supplementary Figure S2. Model validation checks.**

| 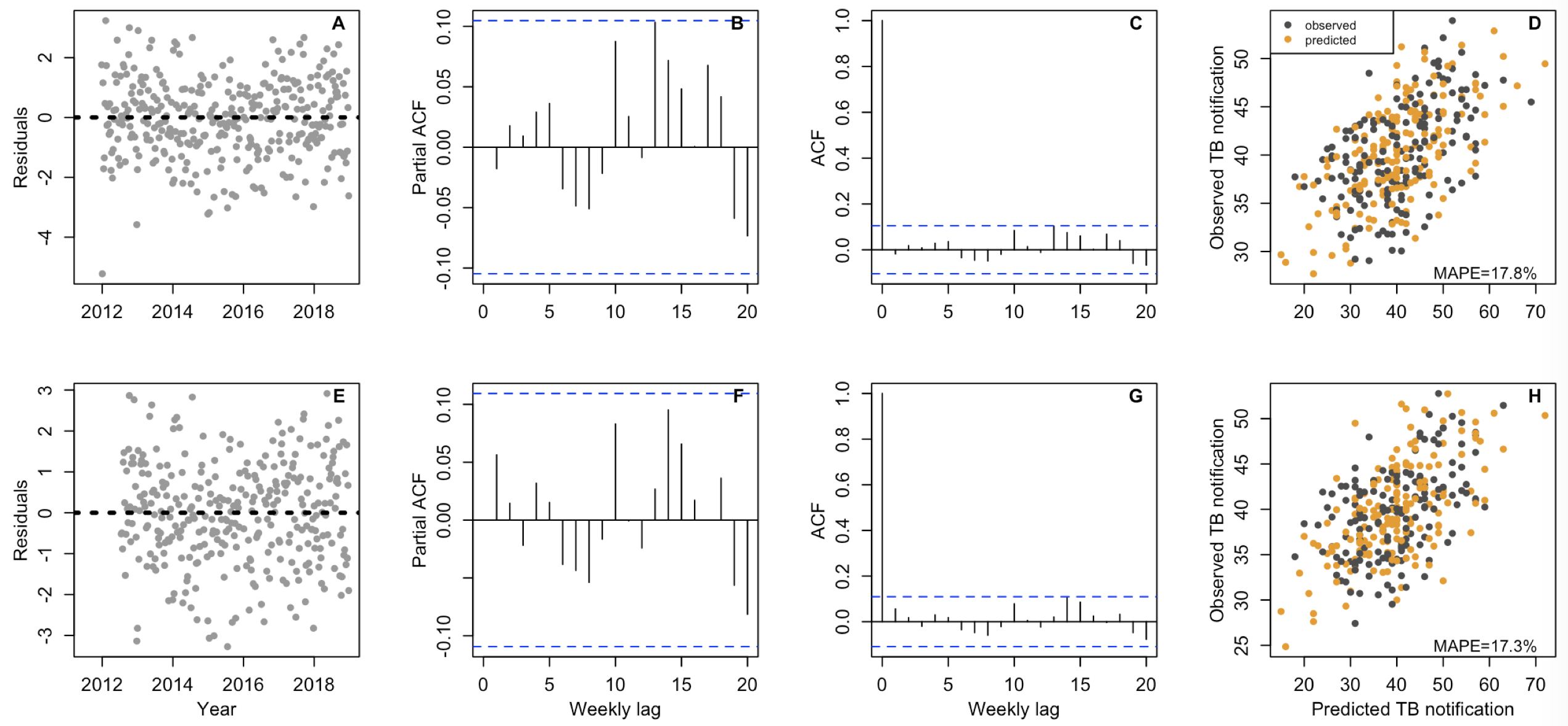  Deviance residuals overtime, partial autocorrelation (partial ACF), autocorrelation (ACF) and model prediction accuracy using mean absolute percentage error (MAPE) respectively, estimated by; adjusted model (additional residuals at lags 1, 2) of bi-dimensional association between rainfall-lag and TB notification rates (A,B,C,D), and adjusted model (additional residuals at lags 2, 13, 17) of bi-dimensional association between temperature-lag and TB notification rates (E,F,G,H). |
| --- |
|  |

| 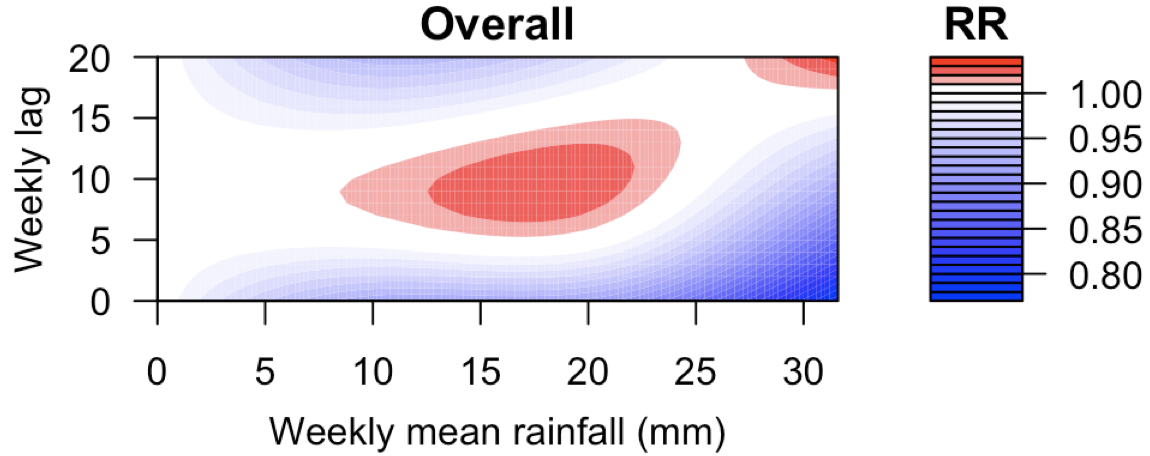 | 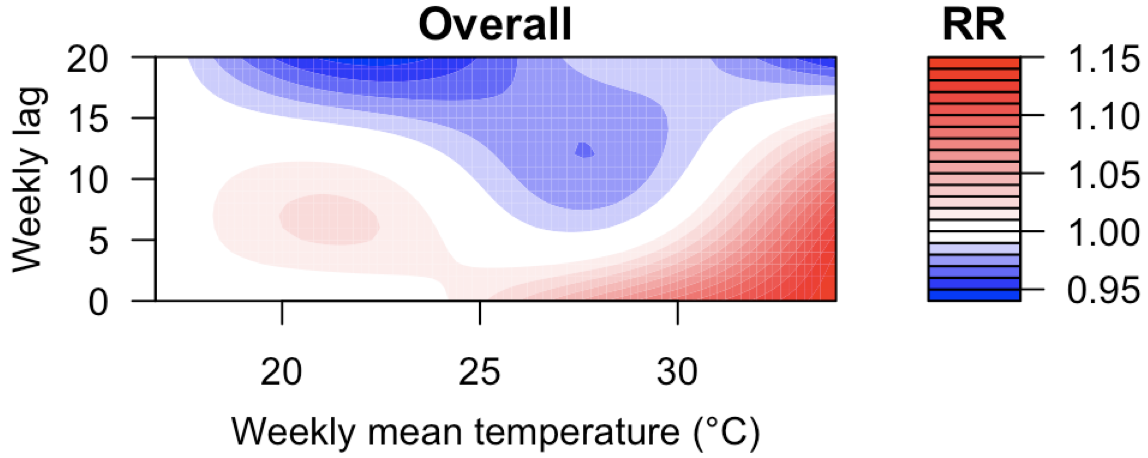 |
| --- | --- |
| 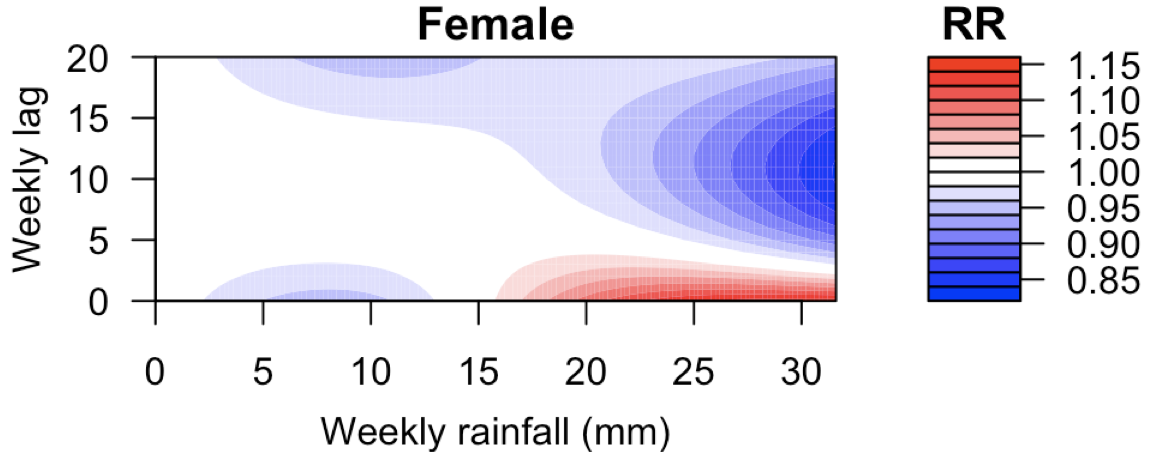 | 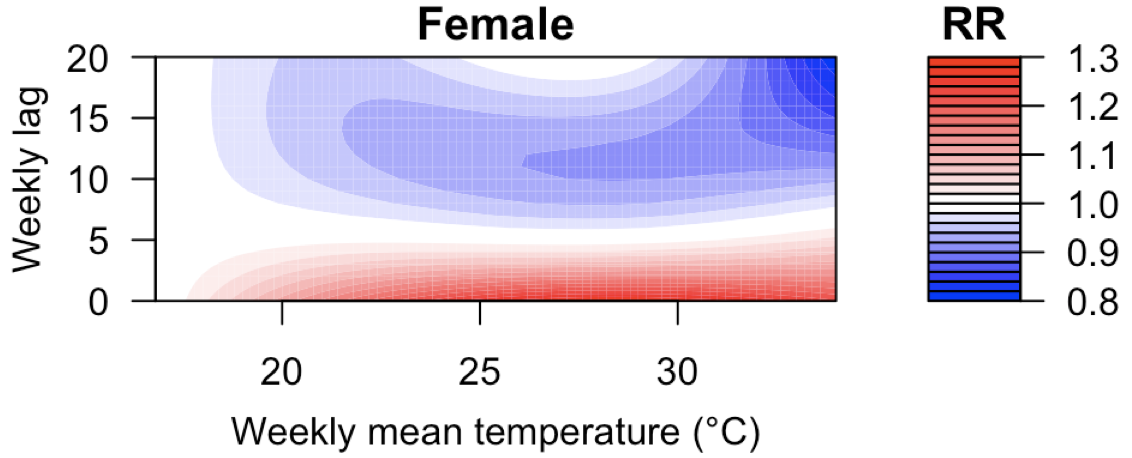 |
| 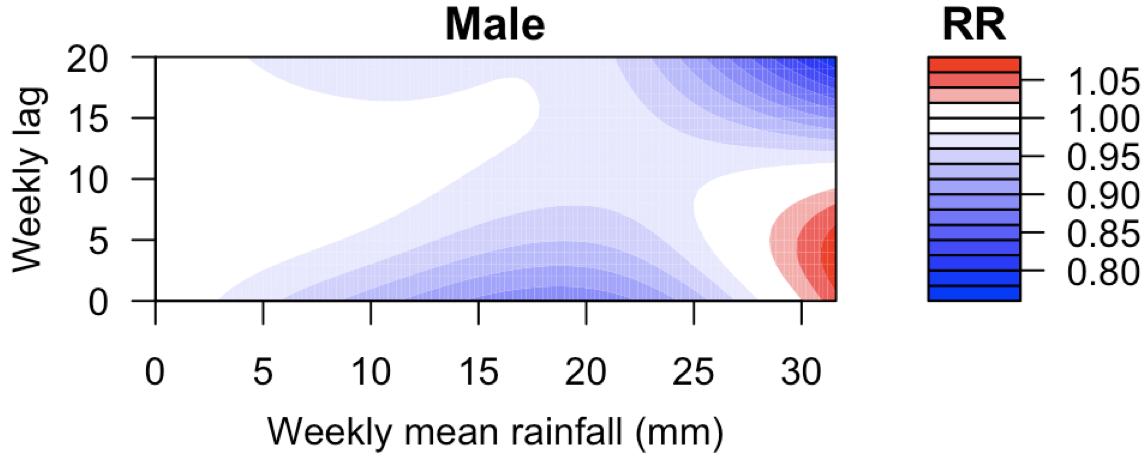 | 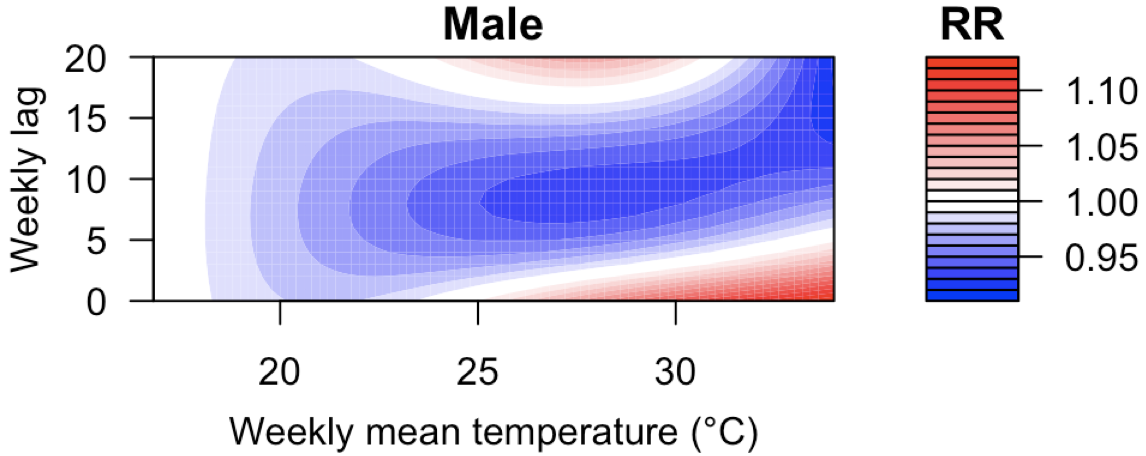 |
| 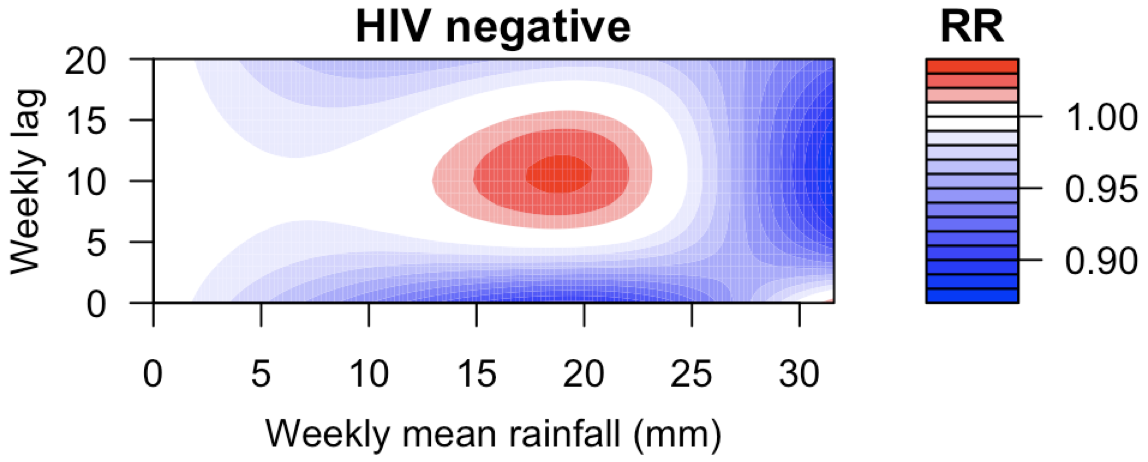 | 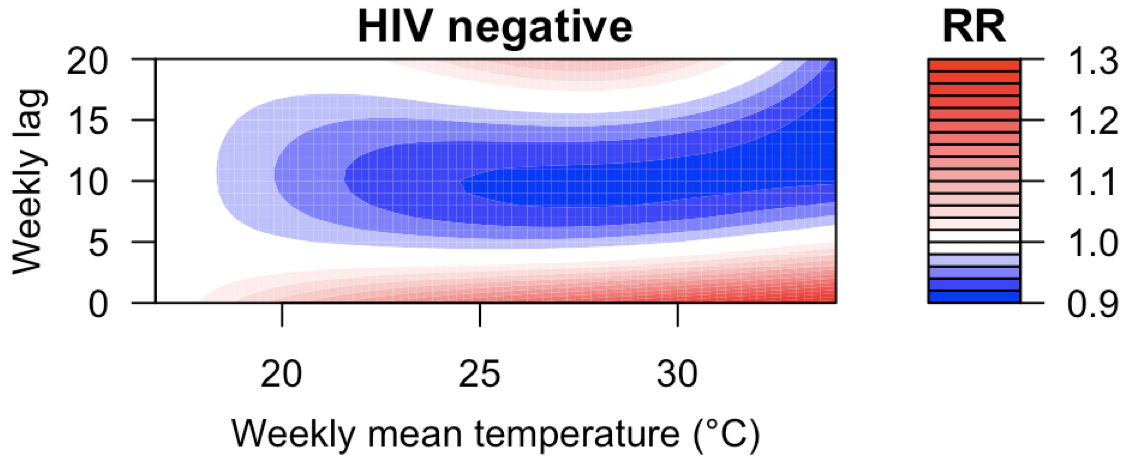 |
| 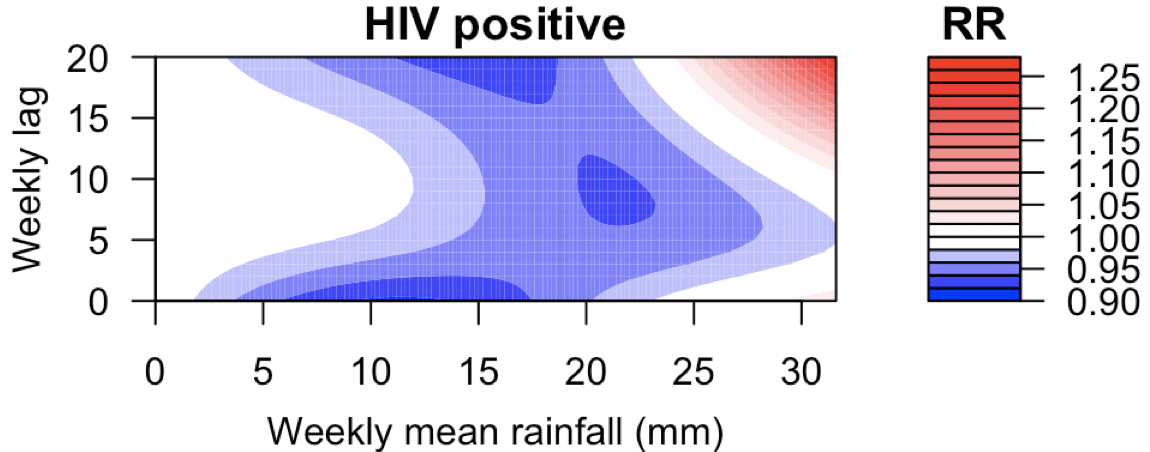 | 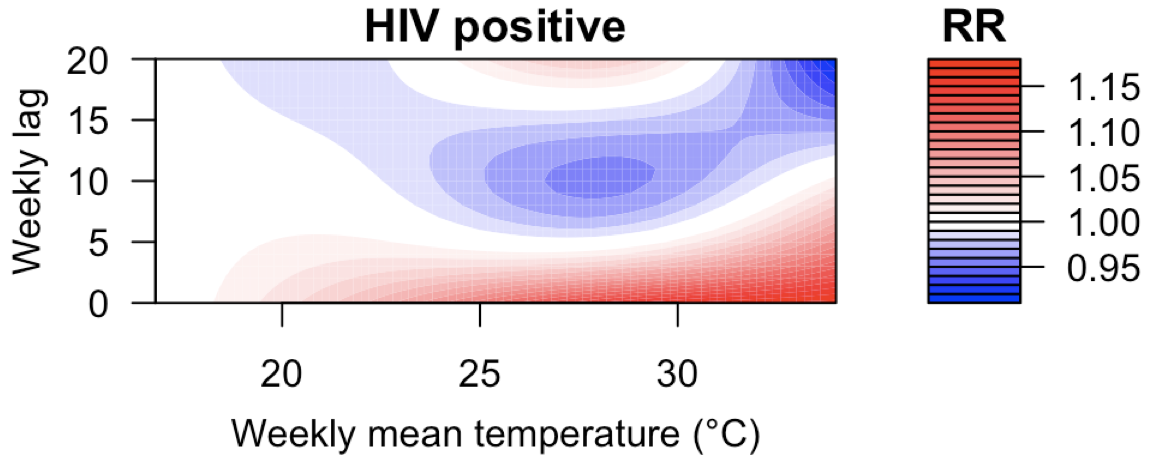 |
| 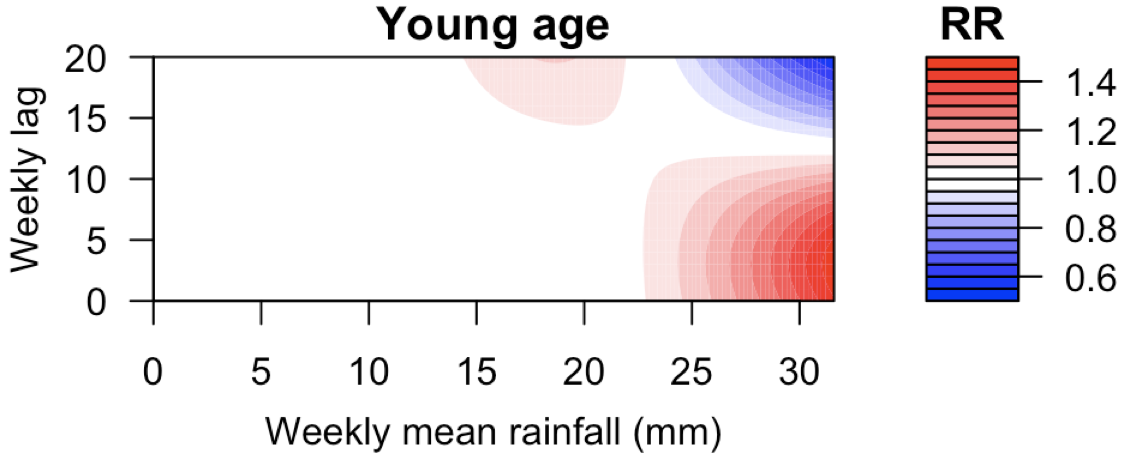 | 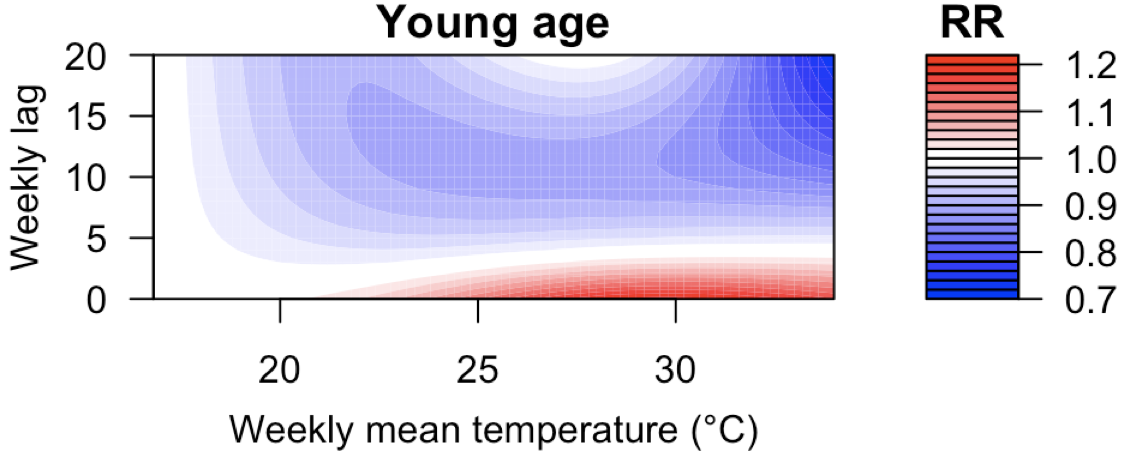 |
| 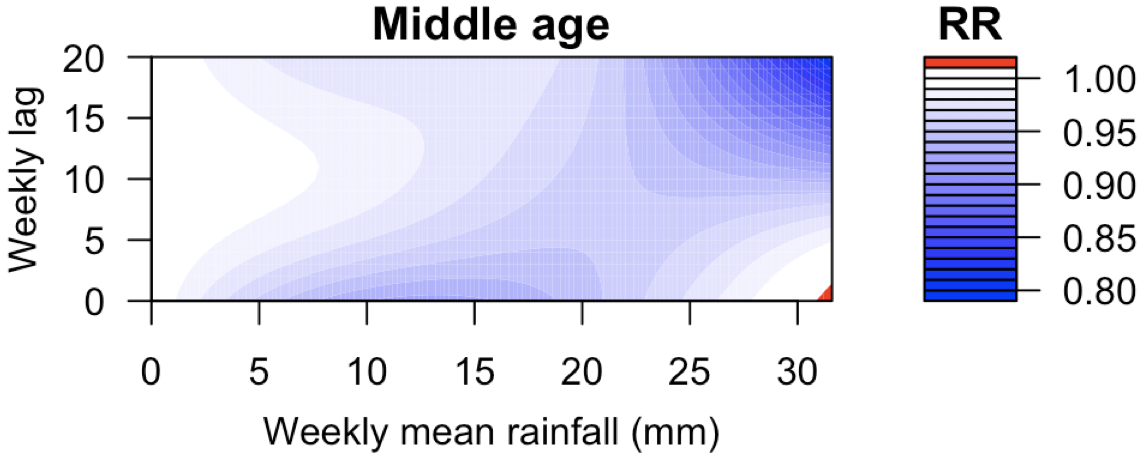 | 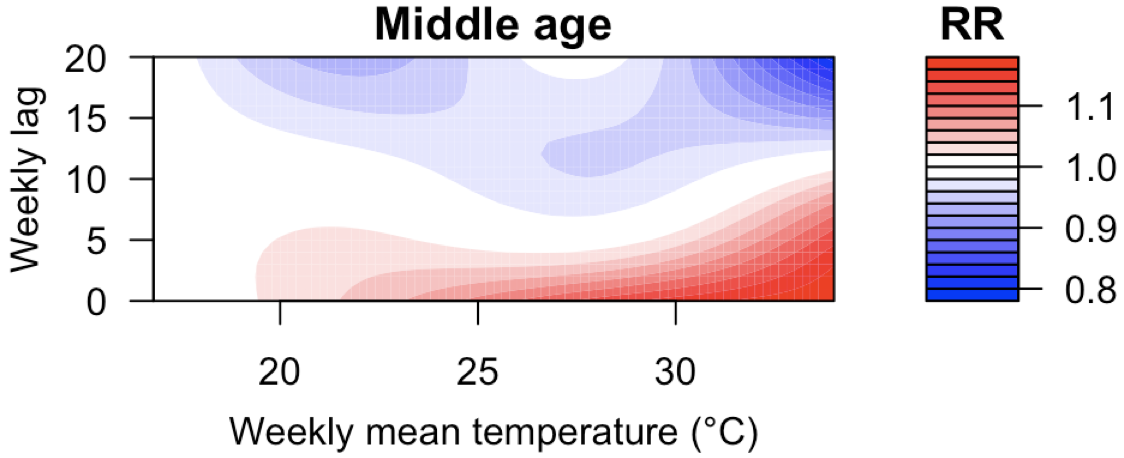 |
| 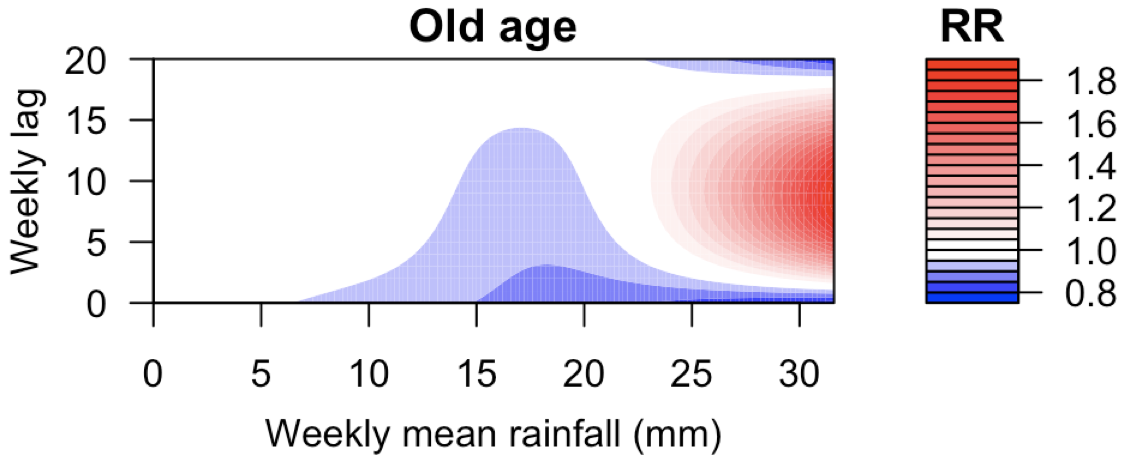 | 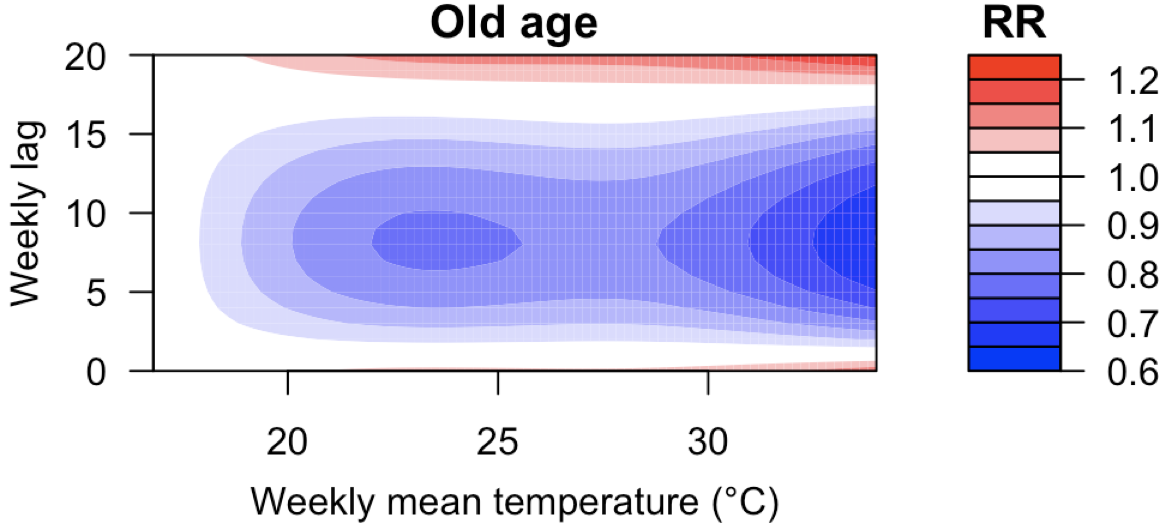 |
| 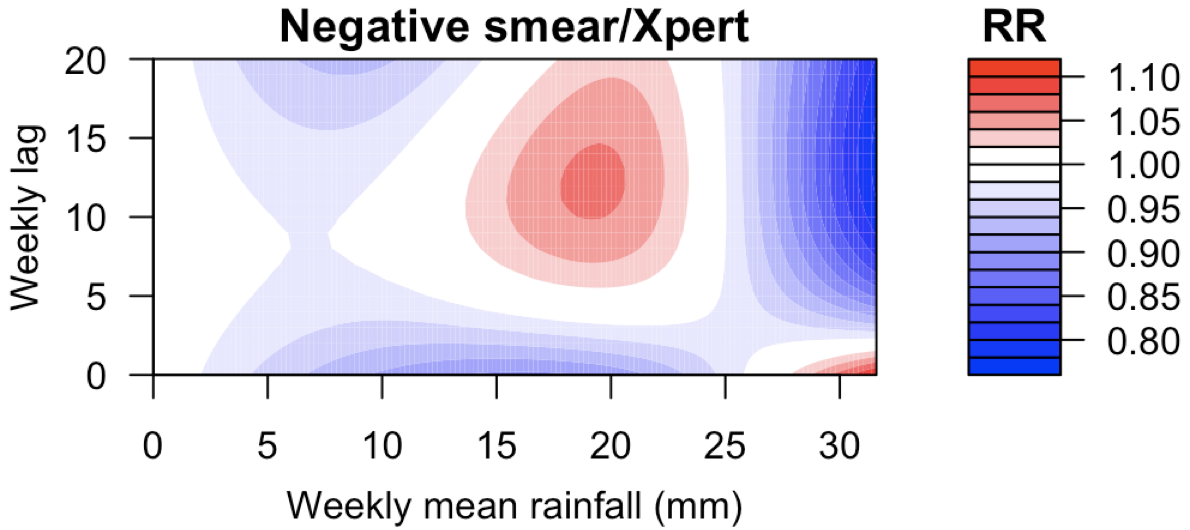 | 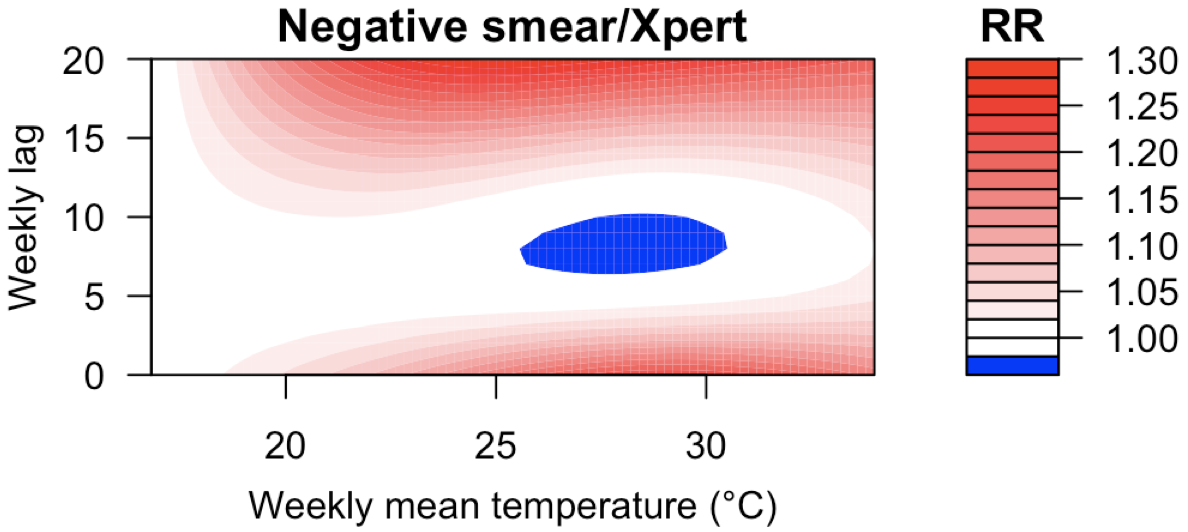 |
| 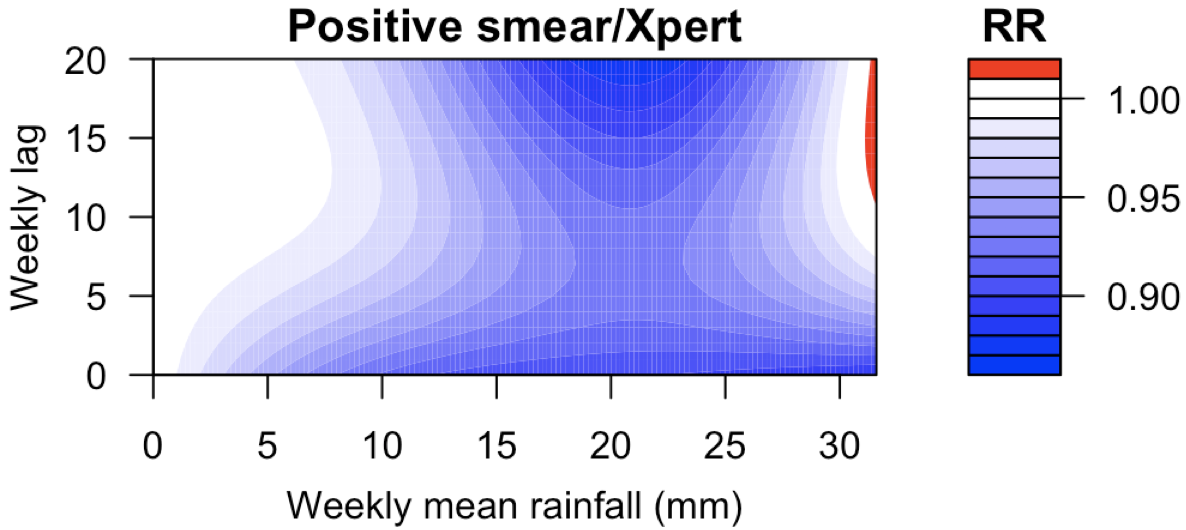 | 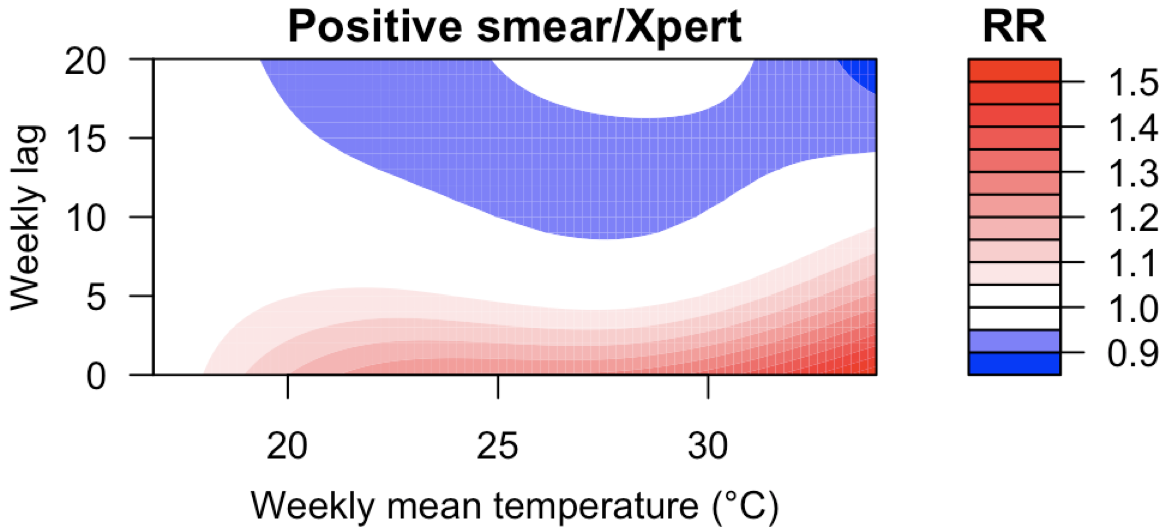 |

**Supplementary Figure S3. Contour plots of the delayed impact of rainfall and temperature on the relative TB notification ratio (RR) stratified by covariates.**
